# Notes and News

**Published:** 1889-03-09

**Authors:** 


### Notes and News.

Collected and Communicated.

In showing a case of leprosy before the Royal Academy of Medicine of Ireland, Dr. Frayer remarked that in olden times leprosy must have been very common in Dublin. Mercer's Hospital used to be a leper hospital, and Lazy Hill evidently took its name from the lazarists who used there to attend to lepers.

The eighteenth congress of the German Surgical Society will be held at Berlin, at Easter, and many of our English surgeons are making plans to spend their Easter holidays in learning what Germany has new to teach them. Prof. von Esmarch, so noted for his various writings and discoveries, will open the congress.

The Museum and Lecture Rooms Syndicate of Cambridge have presented a report proposing to commence the construction of new buildings for human anatomy and physiology, at a cost of £14,000. It is to be hoped the proposal will be carried into effect, as such buildings are badly needed, and would be a great advantage to the many medical students at Cambridge.

The Governors of King's College Hospital held their annual meeting last week. It was reported that this was the jubilee year of the hospital. Last year 2,124 in-patients were admitted to the wards, and 17,635 out-patients treated, the figures showing a large increase of work done over that of 1887. The total expenditure had amounted to £16,901, against £16,534 in 1887.

The Home Secretary has refused to award the Albert medal to Dr. Marcus Eustace, who performed direct transfusion of blood in the case of a woman, a tenant of Lord Ashburton's. The refusal is based on inexpediency of giving the medal to such cases, and the Home Secretary expresses his appreciation of Dr. Eustace's conduct and devotion to a call of professional honour.

Everything is so large in America! The German fair for the New York German Hospital has netted a sum for the institution amounting to £20,000. Now, here is a chance for London to beat its Yankee cousins, and at the forthcoming fancy fair in aid of the Grosvenor Hospital for Women and Children, to spend freely, and we are sure enjoy largely. How surprised and pleased the committee would be if with one effort they cleared £20,000, and were able to start at once on their badly-needed new building!

The Leamington Home for Incurables thinks it ought to receive more support from Leicester. There are six patients within its walls who come from that town, and they each cost £60 a-year, yet Leicester only subscribes £80 a-year to the home. There are 41 patients in the home just now, and an out-patient scheme will shortly be promulgated. It is amusing to notice that the committee never loses a chance of mentioning that their matron is a daughter of Lord Godolphin.

On February 26th the Belfast Royal Hospital held its quarterly meeting. The report said that during the quarter the number of cases treated amounted to 4,950. The intern patients numbered 560, and the extern 4,100. Minor operations to the extent of 150 were performed, so that the demands upon the resources of the institution may not unjustly be considered excessive. We regret, however, to find the hon. secretary calling attention to the backward condition in which the finances of the hospital are at present.

Under a truly characteristic title—"There are others waiting"—the *New York Herald* tells of a method of benefiting hospitals which is simple and excellent. At Huddersfield Infirmary, a mysterious parcel, the outside of which was composed of dress-lining, was found by the porter, and, on being opened, displayed to the gaze of the astonished officials 225 sovereigns. A few years ago a similar mysterious and handsome gift was deposited in the same curious way, the amount on that occasion being £284. It is thought that the two gifts come from the same eccentric but generous donor.

The will of Mr. George Simon, late of Water Lane and Lower Thames Street, and Widmore Lodge, Bromley, wine and spirit merchant, has been proved. The testator bequeaths £50 each to the East London Hospital, the City Mission, and the Homes for Little Boys, Farningham; £25 each to the Governesses' Benevolent Institution, the Dental Hospital, St. Thomas's Hospital, the London Hospital, the Discharged Prisoners' Relief Committee, the City of London Truss Society, the Widmore Schools, the Christ Church Schools, Chislehurst, the Surgical Aid Society, the London Female Preventive Society, Euston Road, and the Bromley Cottage Hospital.

The quarterly general court of governors of the Middlesex Hospital was held on February 28th, when a good report was submitted. Over 30,000 out-patients, and nearly 3,000 in-patients were treated in 1888. Legacies were fairly numerous last year, there was one over £2,000, two over £1,000, and two of £1,000, several smaller legacies, and a special legacy from Miss Cavendish Bentinck of £2,500 towards the Cancer Fund. The Trained Nurses' Institute seems to be answering well, and a balance of £742 has been transferred to capital. This is one of the rare institutions where the nurses get a commission on their earnings. The Samaritan Fund amounted to £636 last year, of which £65 was spent in subscriptions to convalescent homes. All the accounts are audited by chartered accountants.

The City Surveyor and Medical Officer of Health have reported to the Liverpool Hospitals Committee on the Edge Lane Hall Estate. The surveyor estimates the cost of erecting the proposed hospital buildings and boundary walls, forming footpaths, soiling, draining, etc., at £30,500. For the accommodation of the same number of patients in temporary one-storey pavilions—of which double the number would be required—he estimates the cost of the buildings constructed of brick, the administrative block being of a permanent character as proposed in the first scheme, at £27,300. In his second report, which deals with the erection of moveable huts or tents on the estate to provide for the event of an epidemic, the surveyor estimates that to provide for 180 patients eighteen tents would be required, the cost of which, with the necessary boundary walls, draining, wooden buildings, etc., he puts down at £15,700. He adds, however, that when visiting the "Darenth Camp" in company with the chairman of the committee and the medical officer of health, they were informed that the experiment had been anything but successful, and that during one particularly stormy night a panic had seized many of the patients, with the result that they could not be restrained from rushing into the open air. Arrangements of a similar description tried at the Belvidere Hospital, Glasgow, were quickly abandoned.

**The following vacancies are announced this week, the amount of salary in each case being given after the name of the institution :—**

**Resident Medical Officers.**

Rotherham Hospital. Assistant House Surgeon.—Board, etc.
Consumption Hospital, Ventnor. Dispenser.—£60, board, etc.
Brompton Consumption Hospital. Medical Officer. — £200, board, etc.
Birmingham Eye Hospital. Assistant House Surgeon.—£50, board, etc.
Liverpool Northern Hospital. Assistant House Surgeon.—£70, board, etc
Dundee Royal Lunatic Asylum. Medical Assistant.—£80, board, etc.

**Non-resident Medical Officers.**

Dental Hospital of London. Assistant Surgeon.
Guy's Hospital. Six Assistant Dental Surgeons. Lecturer on Dental Anatomy and Physiology, an Anæsthetist, and a Tutor.
St. Saviour's Union, Surrey. District Medical Officer.—£130.
University of Glasgow. Four Examiners in Medicine.—£30.
Brompton Consumption Hospital. Assistant physician.
Surrey Dispensary, Southwark. Dispenser.—£120.
King's College, London. Professor of Forensic Medicine.
Durness, Sutherland. Medical Officer.—£150.
Parish of Paddington. Two Medical Officers.—£75.

At a late meeting of the governors of the Devon and Exeter Hospital, another £700 was voted towards the extensive alterations now being carried on. Several items had been omitted or forgotten in the first estimate, hence the demand for a second supply of funds. The Nurses' Home is to be built on the bit of ground known as the Matron's Garden, and is to cost £1,350. The very faulty system of nursing has been before the Weekly Board for many years, and once proper rooms are provided for the nurses a better class of women may be expected to take these posts, and thorough training will be given to all. The number of both in and out patients is largely increasing, and an urgent appeal to the public for further support must be made.

To secure admission into a hospital at any price seems to be as common in Austria as it is here. A man lying dangerously ill in a village near Carlsbad confessed to a priest that he had recently committed murder in the woods near Meyerling, the scene of the catastrophe in the Imperial family of Austria. The father confessor had no difficulty in inducing the apparently penitent man to repeat his statement before a judicial commission, whereupon the suspect was at once removed to the hospital at Carlsbad. The affair caused a great sensation, and the wildest rumours were set afloat, connecting it with the death of Crown Prince Rudolph. It is now said that the statement is devoid of foundation, and that the alleged murderer, who is in a dying state, has already confessed that he told an untruth for the mere purpose of spending the last days of his life in the more comfortable quarters of Carlsbad Hospital.

The annual general meeting of the governors of the Liverpool Dispensaries was held last week. The Mayor (Mr. E. H. Cookson) presided. The committee reported that the number of patients treated at the North Dispensary in 1888 was 17,847, South 16,204, and East 27,494; total, 61,545 cases, a decrease of 6,781 as compared with 1887. The average number of patients in daily attendance at the dispensaries and patients visited was 641. The decrease in the number of cases is supposed by the committee to be attributable to the general improvement in the public health, and the absence in 1888 of any very serious epidemics. The income for the year was £4,115, of which £1,313 was received in subscriptions, £63 in donations, £150 in legacies, £920 in patients' pence, and £647 from the Hospital Sunday grant. The expenditure was £4,421, and the year closed with a debit balance of £892. Stanley Hospital, Liverpool, also reports a decrease of patients, probably due to the improved sanitation of the town.

A house of rest for women in business must be a useful institution, and a correspondent has sent in a description of a very pretty one at Babbacombe, Devonshire. Here during the summer months some forty women found change and rest, played tennis, learnt to swim and to row, and generally enjoyed a merry and healthy holiday. In the winter many delicate women avail themselves of this home, and coughs depart in a marvellous manner when London fogs are exchanged for the climate of Babbacombe. Admission is by subscriber's ticket and payment of 5s. a-week; or women of business can go as visitors, paying 12s. a-week.

A dramatic entertainment in aid of the Italian Hospital—a hospital open to all nationalities without distinction of religion—was given at the Queen's Gate Hall on Wednesday evening. Two novelties were furnished for the occasion. "Our Family Motto" and "Un Héros de La Vendée," both of them one-act pieces, and from the pen of Miss Heloise Durant. This little play, which was received with favour, was followed by a musical interlude, to which Madame Edith Wynne and Mrs. Godfrey Pearse contributed, Mr. Arthur Helmore giving a very humorous recitation. "Un Héros de La Vendée," which is written in French, served to show Miss Durant's versatility, a pretty idea being carried out with considerable ingenuity. The little hall was well filled, and we hope a substantial sum has been added to the funds of the hospital.

The will of the late Mr. John Rylands, of the firm of Messrs. John Rylands and Sons (Limited), Manchester, who died at Longford Hall, Stratford, on December 11th, has been proved in the Manchester Court of Probate at £2,574 922. Legacies in shares of the firm of Messrs. Rylands and Sons (Limited), representing in paid-up capital, or money paid in advance of calls, or both, the amounts specified, have been left to the undermentioned hospitals: Manchester Royal Infirmary, £5,000; Manchester Royal Eye Hospital, £5,000; Manchester Lock Hospital, £5,000; Wigan Royal Albert Edward Infirmary, £5,000; Manchester and Salford Asylum for Female Penitents, £5,000; St. Mary's Hospital, Manchester, £1,000; and Clerical Hospital for Children, Manchester, £1,000. The total amount of shares bequeathed in charities is £162,000.

Here is an excellent notion from Chester! At the annual and general meeting of the Chester District Working Men's Hospital Saturday Committee recently a project was set on foot to raise funds for a challenge cup, value thirty guineas, to be competed for by the football clubs in Chester in the hope that the amount realised from the "gates" would augment to a considerable extent the subscriptions raised by the committee for the institution. Mr. Sam Laing, one of the committee, wrote soliciting the aid of the Member of Parliament for the city, and Mr. Yerburgh's reply was in the following gratifying terms: "Sir,—I think it an excellent idea of the Working Men's Hospital Saturday Committee that a cup should be given to be played for by the various football clubs in the town which avail themselves of the benefits of Chester Infirmary, and if the committees will permit me I should be glad to have the pleasure of presenting the cup. This, I suggest, shall be a challenge cup to be held for a year by the club winning it, the name of the winner in each year to be engraved upon it. The absolute ownership of the cup I should propose to vest in the Chester District Working Men's Hospital Saturday Committee. Trusting my offer may meet with the approval of the committee, I am, yours faithfully, Robt. Yerburgh." Before receiving Mr. Yerburgh's generous offer, the committee had a good number of subscriptions in hand, and this amount, together with any other sums which may be handed to the committee, will probably be devoted to providing a cup to be competed for by the junior teams in the city and district. We feel sure this plan will provide both funds and patients for the hospital, for the accidents at football seem to grow more numerous daily.

